# Correction of hematopoiesis in alcoholism by the lymphocytes, modulated with a synthetic GABAA-R ligand

**DOI:** 10.1192/j.eurpsy.2025.2031

**Published:** 2025-08-26

**Authors:** E. Markova, I. Savkin, O. Anikeeva, M. Knyazheva, I. Orlovskaya

**Affiliations:** 1 Neuroimmunology Lab; 2 Molecular pathology Lab, State Research Institute of Fundamental and Clinical Immunology, Novosibirsk, Russian Federation

## Abstract

**Introduction:**

It is well known that alcohol has a variety of pathologic effects on hematopoiesis. Long term alcohol abuse result in a significant suppression of both the production of blood cells and structural changes in precursors, namely the suppression of their maturation, up to pancytopenia. We first demonstrated the immunomodulatory properties of a synthetic GABAA-R ligand, meta-chlorobenzhydrylurea (m-CBU). We also showed that splenic lymphocytes modulated *in vitro* by m-CBU after intravenous administration to syngeneic long-term alcoholized recipients have a positive effect, manifested in the editing of behavior characteristic of alcoholism against the background of stimulation neuroplasticity and reduction of neuroinflammation.

**Objectives:**

The purpose of this work was to study bone marrow hematopoiesis and peripheral blood parameters in long-term alcoholized recipients after transplantation of syngeneic lymphocytes modulated in vitro by m-CBU.

**Methods:**

Male (CBAxC57Bl/6)F1 mice with 6-month 10% ethanol exposure were undergoing the transplantation of syngeneic long-term alcoholized mice lymphocytes, pretreated *in vitro* with m-CBU. The number of bone marrow hematopoietic progenitors and cellular composition of the blood were assessed in the recipients.

**Results:**

Long-term alcoholization in mice led to a decrease in the colony-forming activity of hematopoietic precursors, mainly erythroid; in the peripheral blood of mice, a significant decrease in the number of erythrocytes, leukocytes, lymphocytes and platelets was recorded, while the population of segmented neutrophils significantly increased. Lymphocytes, precultured with m-CBU, in syngeneic long-term alcoholized recipients had a corrective effect on a number of hematopoietic parameters (colony-forming activity of erythroid precursors in the bone marrow, the number of erythrocytes, segmented neutrophils and lymphocytes in the peripheral blood) to indicators comparable to those in intact mice of the corresponding with tendency to increase the number of platelets in the peripheral blood.

**Conclusions:**

The data obtained may indicate the effectiveness of m-CBU -modulated lymphocytes transplantation in correcting a number of changes in hematopoiesis provoked by long-term alcohol abuse.

**Disclosure of Interest:**

None Declared

